# Lomerizine Prophylaxis for Pediatric Migraine: Efficacy and Predictors of Treatment Response—A Retrospective Cohort Study

**DOI:** 10.3390/medsci14040447

**Published:** 2026-07-29

**Authors:** Hideki Shimomura, Sachi Tokunaga, Naoko Taniguchi, Eisuke Terasaki, Saeka Yoshitake, Ayuko Usami, Shintaro Iwamoto, Yasuhiro Takeshima

**Affiliations:** 1 Department of Pediatrics, School of Medicine, Hyogo Medical University, 1-1 Mukogawa, Nishinomiya 663-8501, Hyogo, Japan; 2 Department of Pediatrics, Kawanishi City Medical Center, 1-4-1 Hiuchi, Kawanishi City 666-0017, Hyogo, Japan

**Keywords:** migraine, pediatric, lomerizine, preventive therapy, comorbid conditions, psychosocial factors, treatment response

## Abstract

**Background/Objectives:** There is limited evidence supporting the use of lomerizine, a calcium channel blocker approved for migraine prophylaxis in Japan, in children. This study evaluated the prophylactic efficacy of lomerizine in a pediatric population with migraine and identified the baseline factors independently associated with treatment response. **Methods:** This single-center, retrospective cohort study enrolled 83 patients aged ≤18 years prescribed lomerizine for migraine prophylaxis. The primary outcome was headache improvement, defined as a ≥50% reduction in monthly headache days from baseline, assessed at 3–4 and 6–8 months. Multivariable logistic regression included prespecified comorbidity-based covariates: psychosocial factors, neurodevelopmental disorder, and postural orthostatic tachycardia syndrome. **Results:** Headache improvement was observed in 34 of 80 (42.5%) patients at 3–4 months, and in 36 of 71 (50.7%) patients at 6–8 months. Response rates at 3–4 months and 6–8 months, respectively, were 57.7% and 67.4% among patients without psychosocial factors and 14.3% and 20.0% among those with psychosocial factors. Psychosocial factors were the only variables independently associated with a lower probability of improvement at both time points (3–4 months: adjusted odds ratio, 0.122; 95% confidence interval, 0.036–0.419; *p* < 0.001; 6–8 months: adjusted odds ratio, 0.137; 95% confidence interval, 0.041–0.456; *p* = 0.001). **Conclusions:** Patients receiving lomerizine experienced clinically meaningful improvement in headache frequency, particularly those without psychosocial comorbidity. Psychosocial comorbidity was the only baseline factor independently associated with a poor treatment response, underscoring the potential importance of routine psychosocial screening and a possible benefit from concurrent psychological interventions; these findings require prospective confirmation.

## 1. Introduction

Children with migraine often experience emotional and cognitive symptoms, including anxiety, depression, and executive dysfunction, with nearly half experiencing restrictions in daily activities and reduced health-related quality of life [1,2]. Migraine affects the entire household, beyond the individual child, and caregiving has been associated with reduced parental workforce participation [3,4]. These findings underscore the need for effective management strategies in this population.

Pediatric migraine prevention trials are difficult to interpret because treatments do not outperform placebos in most randomized drug trials, even when children show clinical improvements [5,6]. The Childhood and Adolescent Migraine Prevention (CHAMP) trial demonstrated that neither amitriptyline nor topiramate was superior to placebo in reducing headache frequency or disability, a result largely attributed to an unexpectedly high placebo response [7]. The main recurring reasons are high placebo responses, heterogeneous endpoints, pediatric-specific diagnostic and trial design issues, and a thin evidence base that relies heavily on off-label extrapolation from adults [5,8]. Given these limitations, current guidelines recommend a cautious and individualized approach to preventive pharmacotherapy for pediatric migraine, emphasizing the identification of factors associated with treatment response, longitudinal assessment of headache frequency, and careful determination of appropriate treatment duration [5].

Before pharmacological prophylaxis is initiated, non-pharmacological interventions, including lifestyle modification, improving sleep hygiene, and cognitive-behavioral therapy, are recommended as first-line approaches, with behavioral strategies demonstrating efficacy in reducing headache frequency and disability in children [9,10]. Preventive medications are considered when these measures are insufficient. Among these, a recent network meta-analysis identified flunarizine as one of the most effective agents for reducing headache frequency in pediatric migraine [11]. Flunarizine is not approved for use in Japan, where lomerizine hydrochloride is used in its place. Lomerizine hydrochloride is a structurally related agent that shares T-type calcium channel blocking, antihistamine, and anti-serotonin properties with flunarizine but lacks its anti-dopaminergic effect [12]. Lomerizine is approved in Japan for migraine prophylaxis in adults. According to the manufacturer’s package insert, adverse effects reported in pre-approval clinical trials include somnolence, dizziness, and gastrointestinal symptoms such as nausea and constipation, occurring in approximately 11% of patients [13,14]. In contrast to flunarizine, lomerizine has not been associated with sedation; weight gain; depression; extrapyramidal symptoms; or changes in blood pressure, heart rate, or plasma prolactin levels during long-term administration [12,15]. Clinical evidence for the role of lomerizine in migraine prophylaxis is limited primarily to adult studies. In a 6-month open study, lomerizine reduced monthly headache frequency by a mean of 55.2%, with 71% achieving at least a 50% reduction; younger age was independently associated with a better response, suggesting potentially favorable efficacy in pediatric populations [15]. A separate retrospective study identified comorbid tension-type headache and high baseline headache frequency as independent predictors of nonresponse [16]. None of the studies was designed to evaluate lomerizine in children.

The efficacy of preventive pharmacotherapy is not uniform across patients, and comorbid conditions are under-recognized contributors to treatment resistance. Neurodevelopmental disorders, psychosocial comorbidities, and postural orthostatic tachycardia syndrome (POTS) are frequently encountered in children with headaches and are associated with more refractory clinical courses [17,18]. A retrospective study demonstrated that comorbid neurodevelopmental disorders and orthostatic intolerance were independent predictors of nonresponse to prophylactic therapy for pediatric headache [19]. Validated screening tools have shown that brief biopsychosocial risk stratification is feasible at the initial consultation and can identify patients at risk of poor outcomes [20,21]. Despite this evidence, systematic data on how specific comorbidities modulate responses to individual preventive agents, including lomerizine, remain scarce. This study aimed to evaluate the efficacy of lomerizine as a prophylactic agent in a pediatric cohort and identify baseline clinical factors, with a particular focus on comorbid conditions that are independently associated with treatment response.

## 2. Materials and Methods

### 2.1. Study Design and Participants

This was a single-center, retrospective cohort study. We identified all patients aged 18 years or younger who visited the Department of Pediatrics, Hyogo Medical University School of Medicine, between January 2012 and December 2025 and were prescribed lomerizine for migraine prophylaxis. When clinically indicated, magnetic resonance imaging and laboratory tests were performed to exclude secondary headache etiologies, and patients with secondary headache disorders were excluded. Because lomerizine is indicated specifically for migraine prophylaxis, patients whose primary diagnosis was another primary headache disorder, including chronic tension-type headache, rather than migraine, were not a part of this cohort. Headache diagnoses were made in accordance with the criteria of the International Classification of Headache Disorders, Third Edition [22]. All headache evaluations were performed by a single specialist. At the initial consultation, guidance on migraine management and lifestyle modifications was provided to all patients, and additional pharmacological treatments, including lomerizine, were implemented based on individual clinical status. Lomerizine was initiated at a total daily dose of 5 mg (in two divided doses) in patients younger than 10 years and 10 mg (in two divided doses) in patients 10 years or older. If no improvement was observed after approximately 1 month, the dose was increased stepwise, up to a maximum of 20 mg/day, at the treating physician’s discretion, rather than according to a fixed titration protocol. Adherence was assessed through patient/caregiver interview at each visit, and lifestyle guidance was reinforced at every subsequent consultation. All medical records were reviewed by a single physician to ensure consistent application of the predefined diagnostic and eligibility criteria.

The study was conducted in accordance with the Declaration of Helsinki and approved by the Institutional Review Board of Hyogo Medical University (approval number: 5388). Because of the retrospective nature of the study, the requirement for informed consent was waived. Instead, study information was made accessible on the institutional website to allow individuals the option to decline participation.

### 2.2. Outcome Measures

The primary outcome was headache improvement, defined as a ≥50% reduction in monthly headache days from baseline, assessed at two time points: 3–4 months and 6–8 months after initiating lomerizine. This threshold corresponded to a 50% response rate, which is the standard efficacy endpoint in international migraine prevention trials. Headache frequency was assessed at the initial consultation using a structured headache questionnaire completed by the patient, caregiver, or both, and at subsequent visits through interviews with the patient, caregiver, or both. Monthly headache days were estimated by recall of the average number of headache days per week over the preceding month multiplied by four; accordingly, the maximum possible value was 28 days per 4-week period. Each endpoint was analyzed on a complete-case basis; patients without a recorded visit at a given time point were excluded from the corresponding analysis.

### 2.3. Clinical Variables

The following baseline variables were extracted from medical records: sex, age at headache onset, age at lomerizine initiation, baseline headache frequency, number of prior prophylactic agents used, and treatment modality (monotherapy vs. concomitant therapy), migraine subtype (chronic migraine [CM], defined as ≥15 headache days per 4-week period at baseline, versus episodic migraine [EM], defined as <15 headache days per 4-week period; and migraine with versus without aura), and migraine duration (the interval between headache onset and lomerizine initiation). The presence of comorbidities, each reported to be associated with headache chronification or refractoriness to prophylactic therapy [19,23,24], was recorded. Neurodevelopmental disorders were defined as a confirmed diagnosis of autism spectrum disorder or attention-deficit/hyperactivity disorder according to the criteria of the Diagnostic and Statistical Manual of Mental Disorders, Fifth Edition [25]. Psychosocial comorbidity was not classified on the basis of subjective complaints of stress by the patient or caregiver alone, as such complaints are common among children and adolescents and would have resulted in substantial over-classification. Instead, in the absence of a standardized psychometric instrument, psychosocial comorbidity was recorded when the treating specialist, based on clinical interviews with the patient and caregiver(s), either incorporated psychological treatment approaches into the patient’s own outpatient management or referred the patient for external psychological counseling, in response to stressors such as school-related stress or bullying, family conflict or dysfunction, or clinically evident anxiety or depressive symptoms. Because these stressors more often became apparent over the course of follow-up, rather than at the initial visit alone, this assessment was based on clinical interviews conducted throughout the observation period. To keep this threshold consistent, the determination was made by a single specialist throughout the study period. POTS was suspected in patients with orthostatic intolerance. An active standing test was performed, and the diagnosis was made based on established pediatric criteria, defined as an increase in heart rate of ≥35 beats/min within 10 min of standing in the absence of orthostatic hypotension [26]. These comorbidities were not mutually exclusive; a single patient may have met the criteria for more than one condition.

### 2.4. Statistical Analysis

Statistical analyses were performed using SPSS software (version 31.0; IBM Corp., Armonk, NY, USA). Continuous variables are presented as medians with ranges and categorical variables as frequencies and percentages. Baseline clinical characteristics were compared between patients with and without headache improvement at the 3–4- and 6–8-month endpoints using the Mann–Whitney U test for continuous variables and the chi-square test or Fisher’s exact test for categorical variables, as appropriate. Comorbid conditions were analyzed individually in the univariable comparisons.

To identify the baseline factors independently associated with headache improvement, multivariate binary logistic regression analyses were performed separately for the 3–4-month and 6–8-month time points. Covariates were selected a priori based on prior evidence and clinical relevance, including psychosocial comorbidities, neurodevelopmental disorders, and POTS. Although age and sex are established epidemiological correlates of migraine, neither variable differed significantly between the improved and non-improved groups in univariable analyses (both *p* > 0.05 at each time point); therefore, they were excluded from the multivariable model to preserve parsimony given the limited sample size. Additional analysis incorporating both variables confirmed that the adjusted odds ratio (aOR) for psychosocial comorbidities remained virtually unchanged, supporting the robustness of the primary model. Each comorbidity was entered as an independent binary variable and all covariates were entered simultaneously. The results were reported as aOR with 95% confidence intervals (CIs). Given the limited sample size, the number of events per variable was approximately 11–12 in both analyses, which is at the lower boundary of conventional adequacy. Accordingly, these regression analyses should be interpreted as exploratory and hypothesis-generating rather than confirmatory.

Two prespecified analyses were conducted to evaluate the robustness of the responder rate estimates against the assumptions underlying the complete-case analysis. First, the six patients who discontinued follow-up after achieving satisfactory improvement were reclassified as responders at 6–8 months, providing an upper bound for the responder rate at that time point. Second, the three patients who discontinued lomerizine owing to adverse effects were reclassified as non-responders at 3–4 months, representing a worst-case scenario with respect to tolerability. Both assumptions were applied simultaneously to assess the combined effect on the 6–8-month estimate. All statistical tests were two-tailed, and *p*-values < 0.05 were considered statistically significant.

## 3. Results

### 3.1. Baseline Characteristics

A total of 83 patients were included in the analysis. The baseline and demographic characteristics of the patients are presented in Table 1. The median age at headache onset was 10 y (range, 3–17 y) and the median age at lomerizine initiation was 13.0 y (range, 7–17 y). Thirty patients (36.1%) were male and 53 (63.9%) were female. The median baseline headache frequency was 28 d per 4-week period (range, 2–28 d). With regard to prior prophylactic agents, 39 patients (47.0%) had received none, 26 (31.3%) had received one, and 18 (21.7%) had received two. Lomerizine was administered as monotherapy in 74 patients (89.2%) and as concomitant therapy in nine patients (10.8%).

At least one comorbid condition was identified in 46 patients (55.4%). Neurodevelopmental disorders were present in 21 (25.3%) patients, psychosocial factors in 29 (34.9%), and POTS in 14 (16.9%). The distribution of the baseline characteristics according to each comorbid condition is presented in Table 1. These comorbidities were not mutually exclusive and their proportions do not sum to 100%.

### 3.2. Headache Improvement and Univariable Comparisons

Of the 83 enrolled patients, three discontinued lomerizine due to adverse effects prior to the 3–4-month assessment (abdominal pain, *n* = 2; dizziness, *n* = 1) and were excluded from all outcome analyses. Outcome data were available for 80 patients at 3–4 months. At 6–8 months, an additional nine patients were unavailable for follow-up: six discontinued clinic visits after achieving satisfactory headache improvement, two were transferred to a psychiatric department, and one discontinued follow-up without explanation. Accordingly, outcome data were available for 71 patients at 6–8 months. Patients with a recorded visit at a given time point did not have missing data.

Headache improvement was observed in 34 of the 80 patients (42.5%) at 3–4 months and in 36 of the 71 patients (50.7%) at 6–8 months. Table 2 compares the baseline characteristics of patients with and without improvement at each time point. At both time points, the age at headache onset, sex, baseline headache frequency, age at lomerizine initiation, number of prior prophylactic agents, and treatment modality did not differ significantly between the improved and non-improved groups (all *p* > 0.05).

Among comorbid conditions analyzed individually, psychosocial factors were significantly more prevalent in the non-improved group at both time points (3–4 months: 85.7% vs. 42.3%, *p* < 0.001; 6–8 months: 80.0% vs. 32.6%, *p* < 0.001). Neurodevelopmental disorders and POTS were not significantly associated with improvement at any time point (neurodevelopmental disorder: *p* = 0.602 at 3–4 months and *p* = 0.107 at 6–8 months; POTS: *p* = 0.373 at 3–4 months and *p* = 0.245 at 6–8 months). Among patients without psychosocial factors, the response rate was 57.7% (30/52) at 3–4 months and 67.4% (31/46) at 6–8 months, compared with 14.3% (4/28) and 20.0% (5/25) in those with psychosocial factors. Migraine subtype (CM vs. EM) and migraine duration did not differ significantly between the improved and non-improved groups at either time point (subtype: 3–4 months, 39.6% vs. 48.1%, *p* = 0.484; 6–8 months, 47.8% vs. 56.0%, *p* = 0.621; duration: *p* = 0.385 and *p* = 0.508, respectively). Migraine with aura (*n* = 10) showed a numerically higher response rate than migraine without aura at both time points (3–4 months: 70.0% vs. 38.6%, *p* = 0.088; 6–8 months: 87.5% vs. 46.0%, *p* = 0.055), though this difference did not reach statistical significance.

### 3.3. Multivariable Logistic Regression Analysis

The results of the multivariate logistic regression analysis are presented in Table 3. Psychosocial factors were the only variables that were independently associated with headache improvement at both time points. At 3–4 months, the presence of psychosocial factors was significantly associated with a lower probability of improvement (aOR, 0.122; 95% CI, 0.036–0.419; *p* < 0.001). A consistent association of similar magnitude was observed at 6–8 months (aOR, 0.137; 95% CI, 0.041–0.456; *p* = 0.001), supporting the robustness of this finding.

Neurodevelopmental disorders were not significantly associated with improvement at either time point (3–4 months: aOR, 1.058; 95% CI, 0.313–3.575; *p* = 0.928; 6–8 months: aOR, 0.639; 95% CI, 0.175–2.331; *p* = 0.498). The point estimate shifted from above unity at 3–4 months to below unity at 6–8 months; however, both estimates were not statistically significant, with wide CIs, reflecting insufficient statistical power attributable to the small number of patients with this comorbidity (*n* = 21). Similarly, POTS was not independently associated with improvement at any time point (3–4 months: aOR, 0.509; 95% CI, 0.130–1.993; *p* = 0.332; 6–8 months: aOR, 0.467; 95% CI, 0.121–1.795; *p* = 0.267), likely because of the small sample size (*n* = 14).

### 3.4. Sensitivity Analyses

Two prespecified sensitivity analyses were conducted to assess the robustness of the responder rate estimates against the assumptions underlying the complete-case analysis. In the first analysis, six patients who discontinued follow-up after achieving satisfactory improvement were reclassified as responders at 6–8 months. Under this assumption, the responder rate at 6–8 months increased from 36 of 71 (50.7%) to 42 of 77 (54.5%), indicating that the complete-case estimate was conservative and that the true responder rate at 6–8 months was likely to be at least as high as the primary estimate. In the second analysis, the three patients who discontinued lomerizine due to adverse effects were reclassified as non-responders at 3–4 months, representing a worst-case scenario with respect to tolerability. Under this assumption, the response rate at 3–4 months decreased from 34 of 80 (42.5%) to 34 of 83 (41.0%). When both assumptions were applied simultaneously, adverse effect discontinuations were classified as non-responders and improvement-related discontinuations were classified as responders; the 6–8-month responder rate was 42/77 (54.5%). Across all scenarios, the direction and approximate magnitude of the responder rate estimates remained consistent, thereby supporting the stability of the primary findings.

## 4. Discussion

This retrospective cohort study showed that headache improvement occurred in 42.5% at 3–4 months and 50.7% at 6–8 months in a pediatric population with migraine. These findings suggest the potential effectiveness of lomerizine for headache prophylaxis in this population, with improvement that was stable from the early to the later assessment period. These figures are broadly consistent with the 71% response rate reported in the only available adult study on long-term lomerizine administration [15], although direct comparisons are limited by differences in study populations, baseline headache frequency, and outcome assessment methods. Among the baseline clinical variables examined, psychosocial comorbidity was the only factor independently associated with treatment response at both time points, with more than sevenfold lower odds of improvement in patients with psychosocial factors (Table 3). The consistency of this association across two independent time points strengthens the inference that psychosocial comorbidity is a robust negative predictor of lomerizine response in this population, rather than a chance finding.

Contextualizing these findings within the broader literature is important, given the well-documented difficulty of demonstrating drug-specific efficacy over placebo in pediatric migraine [5,6]. In the CHAMP trial, 50% responder rates at 24 weeks were 52% for amitriptyline, 55% for topiramate, and 61% for placebo, with no significant difference among groups [7]. A network meta-analysis similarly revealed no treatment more effective than placebo over the long term; flunarizine, the calcium channel blocker most similar to lomerizine, showed the largest point estimate but remained nonsignificant, based on a single small trial [6], consistent with the American Academy of Neurology and American Headache Society (AAN/AHS) guideline, which concluded that evidence supporting flunarizine is insufficient [5]. The overall responder rate observed here (42.5–50.7%) was similar to or lower than CHAMP’s active-drug rates, as well as lower than its placebo rate, reinforcing caution regarding causal interpretation in this uncontrolled study. The markedly higher rate among patients without psychosocial comorbidity (57.7–67.4%) approached CHAMP’s active-treatment response rates, whereas the rates among those with psychosocial comorbidity were substantially lower (14.3–20.0%), consistent with the AAN/AHS recommendations to screen for comorbid mood and anxiety disorders given their association with headache persistence [5].

The finding that psychosocial comorbidity was the only independent predictor of lomerizine response is consistent with a broader literature linking psychosocial comorbidity to poorer headache outcomes irrespective of the pharmacological agent used. Prior studies have associated internalizing and externalizing symptoms, family conflict, and school-related stress with pediatric headache [27], shown a stronger association between maladaptive psychological traits and migraine than tension-type headache [28], and identified dysfunctional coping, rather than the mere presence of emotional symptoms, as an independent predictor of failure to achieve headache remission [29]. Although these constructs differ from the operationalization used in the present study, they converge on the principle that the functional impact of psychosocial burden, rather than its presence alone, is most closely linked to headache outcomes—a conclusion reinforced by a clinic-based study in which higher psychosocial burden predicted a poorer response even to combined pharmacological and psychological treatment [30].

Taken together with our findings, these data suggest that psychosocial comorbidities function as robust modifiers of treatment response across different drugs and clinical settings. The operational definition of psychosocial comorbidity used in this study, requiring psychosocial stressors of sufficient severity to warrant targeted counseling or psychotherapeutic intervention beyond standard lifestyle guidance, was intentionally designed to identify patients with a clinically meaningful psychosocial burden rather than subclinical stress. This threshold may have captured a subgroup in which unresolved psychosocial stressors are associated with headache chronicity, thereby limiting the pharmacological effects of lomerizine. This interpretation is supported by prior work from our institution demonstrating that emotional and behavioral problems identifiable at the first consultation predict treatment outcomes in pediatric headache [31] and that emotional problems, when present alongside neurodevelopmental disorders, add complexity to headache management that pharmacotherapy alone cannot adequately address [32]. Collectively, these observations support incorporating psychosocial assessment into the initial evaluation of children with headache and suggest that patients identified as having a significant psychosocial burden may benefit from concurrent psychological intervention alongside pharmacological therapy, a hypothesis that warrants prospective testing [33,34].

In contrast to psychosocial comorbidities, neither neurodevelopmental disorders nor POTS were independently associated with the lomerizine response at either time point (Table 3). This null finding should be interpreted with caution given the limited statistical power of the present study; the subgroups with neurodevelopmental disorders and POTS were small (Table 1), and with approximately 11–12 events per variable, the analyses were underpowered to detect associations of moderate magnitude. The wide CIs for both variables and the directional instability of the neurodevelopmental disorder estimate across time points (Table 3) reflect sampling uncertainty rather than true effect absence. The broader literature suggests that both conditions are plausible modifiers of the prophylactic response. Headache is one of the most common comorbidities in pediatric patients with POTS, occurring in up to 43% of patients with migraine, and those with unexplained pain show less favorable treatment outcomes overall [35,36]. A systematic review and meta-analysis further confirmed that headache disorders are highly prevalent in patients with POTS, with proposed mechanisms, including orthostatic cerebral hypoperfusion and autonomic dysregulation pathways, which may be only partially amenable to calcium channel blockade [36]. In pediatric patients with POTS and comorbid chronic pain, multidisciplinary management has been advocated because pharmacological monotherapy is insufficient to simultaneously address autonomic and pain-sensitization components [37]. With respect to neurodevelopmental disorders, catecholaminergic dysregulation inherent to attention-deficit/hyperactivity disorder, which shares pathophysiological pathways with pain sensitization [38], and sensory hyperreactivity characteristic of autism spectrum disorder, which amplifies trigeminal nociception through mechanisms distinct from calcium channel blockade [39,40], may independently sustain headache chronicity beyond the pharmacological reach of lomerizine. These considerations, combined with the consistent findings of our previous cyproheptadine study at the same institution [19], suggest that the absence of statistical significance for neurodevelopmental disorders and POTS in the present study reflects insufficient power rather than a genuine null effect. Adequately powered prospective studies are needed to clarify the moderating role of these comorbidities on the response to prophylactic treatment in pediatric migraine. Migraine subtype and duration were not associated with treatment response, reinforcing the specificity of psychosocial comorbidity as a predictor in this cohort. Although the response rate was numerically higher in the small subgroup of patients with aura, the difference did not reach significance and should be regarded as hypothesis-generating, warranting confirmation in larger cohorts.

Among patients without psychosocial comorbidities, the treatment response rates were 57.7% at 3–4 months and 67.4% at 6–8 months in univariable comparisons (Table 2), figures that compare favorably with adult studies and suggest that lomerizine may be particularly effective in this subgroup. Conversely, patients with psychosocial comorbidities achieved improvement in only 14.3% and 20.0% of cases at the respective time points (Table 2). This contrast underscores the clinical value of routine psychosocial screening at the initial consultation. Validated brief tools, such as the Pediatric Pain Screening Tool and the Psychosocial Assessment Tool, have demonstrated that biopsychosocial risk stratification is feasible in pediatric headache clinics and can identify patients who are unlikely to respond to pharmacological treatment alone [20,21,31,34]. Patients identified as having a clinically significant psychosocial burden may benefit from concurrent behavioral or psychotherapeutic interventions targeted at the identified stressors, as behavioral approaches have been reported to enhance or, in some cases, serve as an alternative to pharmacotherapy in other pediatric headache populations [9,10,30,33]. More broadly, these data support an individualized, comorbidity-informed approach to preventive pharmacotherapy in pediatric headache, in which treatment selection is guided not only by headache characteristics but also by the psychosocial and neurodevelopmental profile of each patient [5].

This study had several limitations that should be considered when interpreting the findings. First, as a single-center, retrospective cohort study, it was subject to selection bias and unmeasured confounding factors; the patient population reflected a tertiary referral center and may not be representative of the broader pediatric migraine population. Second, the sample size was modest, and the number of events per variable was at the lower boundary of conventional adequacy, resulting in insufficient statistical power to detect associations of moderate magnitude for neurodevelopmental disorder and POTS; accordingly, the regression analyses should be considered exploratory rather than confirmatory. Third, the operational definition of psychosocial comorbidity, requiring factors of sufficient severity to warrant targeted intervention, was developed for clinical use at our institution and may limit comparability with studies using standardized psychometric instruments, thereby constraining the external validity of this finding. This determination was based on unstructured clinical judgment by a single specialist, rather than a validated psychometric tool, introducing the possibility of observer-dependent variability in the classification of psychosocial comorbidity. Although this may have reduced precision, it would not necessarily alter the direction of the observed association. Furthermore, because psychosocial stressors were sometimes identified during follow-up rather than exclusively at treatment initiation, the temporal separation between this covariate and the outcome assessment was not absolute; this finding raises the possibility that patients with a poorer clinical course were evaluated more closely for psychosocial factors, which could partly reflect detection bias, rather than a purely baseline predictive relationship. Although migraine subtype and duration were examined and did not alter the primary conclusions, medication overuse could not be systematically extracted from the retrospective records and therefore remains an unmeasured confounder. In addition, the number of variables examined raises the possibility that the aura finding reflects chance. Fourth, headache frequency was assessed with a maximum possible value of 28 d per 4-week period, given that the median baseline headache frequency was 28 d—the ceiling of this scale—the true burden may have been underestimated in a substantial proportion of patients, and the 50% response rate may itself be a conservative estimate of the clinical benefit in the most severely affected subgroup. Relatedly, headache frequency was estimated from recall by the patient, caregiver, or both, rather than from prospectively completed headache diaries, introducing the possibility of recall bias and outcome misclassification that could have under- or over-estimated baseline severity and treatment response. Fifth, the absence of a control group precluded the definitive attribution of headache improvement to lomerizine, as spontaneous remission and placebo effects cannot be excluded, particularly given the well-documented high placebo response rates in pediatric headache trials [7]. Sixth, the follow-up period of 6–8 months did not capture long-term efficacy or safety, and the absence of systematic adverse event monitoring and dose–response data limited conclusions regarding the optimal dosing strategy for lomerizine in children. Relatedly, the 28-day recall window also constrained assessment of long-term durability; future prospective studies should extend follow-up and use diaries capturing headache burden over longer intervals.

Despite these limitations, the present study had several strengths. To the best of our knowledge, this is the first dedicated analysis of lomerizine prophylaxis in a pediatric population with migraine, addressing a clinically important evidence gap in a setting where flunarizine is unavailable. Furthermore, the a priori specification of comorbidity-based covariates, the conduct of prespecified sensitivity analyses to assess the robustness of responder rate estimates, and an additional analysis confirming the stability of the primary multivariable model strengthen the analytical rigor of the findings. The consistent application of eligibility and diagnostic criteria by a single headache specialist throughout the study period ensured diagnostic uniformity. However, the absence of independent outcome assessment introduced the possibility of detection bias, as the treating physician was not blinded to the treatment or comorbidity status when evaluating headache improvement.

## 5. Conclusions

This retrospective cohort study provided preliminary evidence that lomerizine led to clinically meaningful improvement in headache frequency in children and adolescents with migraine, particularly in those without psychosocial comorbidity. Psychosocial comorbidity was the only baseline factor independently associated with poor treatment response at both time points, underscoring the potential value of routine psychosocial screening at the initial consultation and suggesting a possible benefit from concurrent psychological intervention in patients with a significant psychosocial burden, a hypothesis that requires prospective confirmation. These findings support an individualized, comorbidity-informed approach to preventive pharmacotherapy in pediatric migraine. Prospective controlled studies with standardized psychosocial assessment tools and adequate statistical power are needed to confirm these findings and clarify the moderating roles of neurodevelopmental disorders and POTS in prophylactic treatment outcomes.

## Figures and Tables

**Table 1:** Baseline and demographic characteristics of the study population.

	Total	Neurodevelopmental Disorder	Psychosocial Factors	POTS	No Comorbidity
Total number of participants, *n* (%)	83	21 (25.3)	29 (34.9)	14 (16.9)	37 (44.6)
Median age at headache onset, y (range)	10 (3–17)	10.0 (3–13)	9 (3–14)	11 (4–14)	10 (3–17)
Sex, *n* (%)					
Male	30 (36.1)	10	7	7	14
Female	53 (63.9)	11	22	7	23
Median age at lomerizine initiation, y (range)	13.0 (7–17)	14.0 (9–17)	13 (8–17)	14 (12–16)	13 (7–17)
Migraine duration, y (range)	3 (0–11)	4 (0–10)	4 (0–10)	4 (0–8)	3 (0–11)
Headache frequency before lomerizine administration, median (range)	28 (2–28)	28 (2–28)	28 (4–28)	28 (12–28)	16 (2–28)
Migraine subtype, *n* (%)					
Chronic migraine	56 (67.5)	15	24	13	20
Episodic migraine	27 (32.5)	6	5	1	17
Migraine without aura	73 (88.0)	20	28	14	29
Migraine with aura	10 (12.0)	1	1	0	8
Number of prior prophylactic agents, *n* (%)					
0	39 (47.0)	8	12	9	18
1	26 (31.3)	7	11	3	12
2	18 (21.7)	6	6	2	7
Monotherapy or concomitant therapy					
Monotherapy	74 (89.2)	18	26	13	34
Concomitant therapy	9 (10.8)	3	3	1	3

Comorbid conditions are not mutually exclusive; percentages do not sum to 100%. Abbreviation: POTS, postural orthostatic tachycardia syndrome.

**Table 2:** Comparison of baseline characteristics between patients with and without headache improvement at 3–4 and 6–8 months.

	3–4 Months			6–8 Months		
	Improved *n* = 34	Not Improved *n* = 46	*p*-Value	Improved *n* = 36	Not Improved *n* = 35	*p*-Value
Age at headache onset, y (range)	9.5 (3–17)	10 (3–15)	0.803	10 (3–17)	10 (3–15)	0.849
Sex, *n* (%)						
Male	13 (44.8)	16 (55.2)	0.816	14 (56.0)	11 (44.0)	0.621
Female	21 (41.2)	30 (58.8)		22 (47.8)	24 (52.2)	
Age at lomerizine initiation, y (range)	12.5 (7–17)	14 (9–17)	0.245	14 (7–17)	14 (10–17)	0.904
Migraine duration, y (range)	3 (0–10)	3.5 (0–11)	0.385	4 (0–10)	4 (0–11)	0.508
Headache frequency before lomerizine administration, median (range)	28 (2–28)	28 (4–28)	0.407	28 (2–28)	28 (3–28)	0.338
Migraine subtype						
Chronic migraine	21 (39.6)	32 (60.4)	0.484	22 (47.8)	24 (52.2)	0.621
Episodic migraine	13 (48.1)	14 (51.9)		14 (56.0)	11 (44.0)	
Migraine without aura	27 (38.6)	43 (61.4)	0.088	29 (46.0)	34 (54.0)	0.055
Migraine with aura	7 (70.0)	3 (30.0)		7 (87.5)	1 (12.5)	
Number of prior prophylactic agents, *n* (%)						
0	16 (41.0)	23 (59.0)	0.898	17 (48.6)	18 (51.4)	0.861
1	12 (46.2)	14 (53.8)		11 (50.0)	11 (50.0)	
2	6 (40.0)	9 (60.0)		8 (57.1)	6 (42.9)	
Monotherapy or concomitant therapy, *n* (%)						
Monotherapy	28 (40.3)	43 (59.7)	0.159	32 (50.0)	32 (50.0)	1.000
Concomitant therapy	6 (66.7)	3 (33.3)		4 (57.1)	3 (42.9)	
Comorbid conditions, *n* (%)						
Neurodevelopmental disorder						
Yes	7 (35.0)	13 (65.0)	0.602	6 (33.3)	12 (66.7)	0.107
No	27 (45.0)	33 (55.0)		30 (56.6)	23 (43.4)	
Psychosocial factors						
Yes	4 (14.3)	24 (85.7)	<0.001	5 (20.0)	20 (80.0)	<0.001
No	30 (57.7)	22 (42.3)		31 (67.4)	15 (32.6)	
POTS						
Yes	4 (28.6)	10 (71.4)	0.373	5 (35.7)	9 (64.3)	0.245
No	30 (45.5)	36 (54.5)		31 (54.4)	26 (45.6)	

Abbreviation: POTS, postural orthostatic tachycardia syndrome.

**Table 3:** Multivariable logistic regression analysis of factors associated with headache improvement.

	3–4 Months			6–8 Months		
Variables	aOR	95% CI	*p*-Value	aOR	95% CI	*p*-Value
Psychosocial factors	0.122	0.036–0.419	<0.001	0.137	0.041–0.456	0.001
Neurodevelopmental disorder	1.058	0.313–3.575	0.928	0.639	0.175–2.331	0.498
POTS	0.509	0.130–1.993	0.332	0.467	0.121–1.795	0.267

Covariates included psychosocial factors, neurodevelopmental disorder, and POTS, entered simultaneously as independent binary variables. Abbreviations: aOR, adjusted odds ratio; CI, confidence interval; POTS, postural orthostatic tachycardia syndrome.

## Data Availability

The original contributions presented in this study are included in the article. Further inquiries can be directed to the corresponding author.

## Abbreviations

The following abbreviations are used in this manuscript:

aOR	adjusted odds ratio
CI	confidence interval
CM	chronic migraine
EM	episodic migraine
POTS	postural orthostatic tachycardia syndrome
